# Plant Proteins for Future Foods: A Roadmap

**DOI:** 10.3390/foods10081967

**Published:** 2021-08-23

**Authors:** Shaun Yong Jie Sim, Akila SRV, Jie Hong Chiang, Christiani Jeyakumar Henry

**Affiliations:** 1 Clinical Nutrition Research Centre (CNRC), Singapore Institute of Food and Biotechnology Innovation (SIFBI), Agency for Science, Technology and Research (A*STAR), Singapore 117599, Singapore; akila_srinivasagam_ramasamy_venkateswaran@sifbi.a-star.edu.sg (A.S.); chiang_jie_hong@sifbi.a-star.edu.sg (J.H.C.); jeya_henry@sifbi.a-star.edu.sg (C.J.H.); 2 Department of Biochemistry, Yong Loo Lin School of Medicine, National University of Singapore, Singapore 117596, Singapore

**Keywords:** plant proteins, future foods, animal alternatives, nutrition

## Abstract

Protein calories consumed by people all over the world approximate 15–20% of their energy intake. This makes protein a major nutritional imperative. Today, we are facing an unprecedented challenge to produce and distribute adequate protein to feed over nine billion people by 2050, in an environmentally sustainable and affordable way. Plant-based proteins present a promising solution to our nutritional needs due to their long history of crop use and cultivation, lower cost of production, and easy access in many parts of the world. However, plant proteins have comparatively poor functionality, defined as poor solubility, foaming, emulsifying, and gelling properties, limiting their use in food products. Relative to animal proteins, including dairy products, plant protein technology is still in its infancy. To bridge this gap, advances in plant protein ingredient development and the knowledge to construct plant-based foods are sorely needed. This review focuses on some salient features in the science and technology of plant proteins, providing the current state of the art and highlighting new research directions. It focuses on how manipulating plant protein structures during protein extraction, fractionation, and modification can considerably enhance protein functionality. To create novel plant-based foods, important considerations such as protein–polysaccharide interactions, the inclusion of plant protein-generated flavors, and some novel techniques to structure plant proteins are discussed. Finally, the attention to nutrition as a compass to navigate the plant protein roadmap is also considered.

## 1. Introduction

With global populations projected to increase above nine billion people by 2050 [1], we face an unprecedented challenge to produce and distribute adequate food to all of mankind. Apart from first meeting our calorie needs, the second most important macronutrient needed for human survival is protein. Protein production is a major concern because traditional animal protein sources require an intensive amount of land and resources [2]. Plant-based proteins represent a promising solution due to their long history of crop use and cultivation, lower cost of production, and easy access in many parts of the world. Plant proteins are also more environmentally sustainable [3]. However, in addition to lower protein quality, plant proteins also have comparatively poor functionality, defined as poor solubility, foaming, emulsifying, and gelling properties, limiting their use in food products. Relative to animal proteins, including dairy products, plant protein technology is still in its infancy. To bridge this gap, advances in plant protein ingredient development and the knowledge to construct plant-based foods are sorely needed.

**Figure 1:**
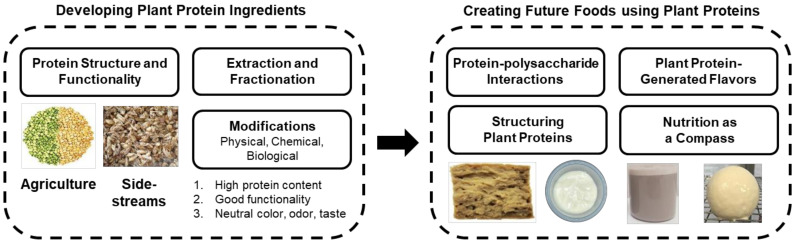
A roadmap to accelerate plant protein science and technology, focusing on plant protein ingredient development and future food creation.

This review is an attempt to stimulate interest and presents a roadmap to accelerate plant protein science and technology, focusing on plant protein ingredient development and future food creation (Figure 1). In each area, the current state of the art is briefly presented, and new research directions are highlighted. The purpose of this review is not to replicate what has been done, but to inject fresh ideas and to foster new thinking. Readers interested in more detailed discussions about various plant protein topics are referred to prior excellent reviews [4,5,6,7,8,9]. This paper focuses on manipulating plant protein structures during (a) protein extraction, (b) fractionation, and (c) modification. To create novel plant-based foods, important considerations such as protein–polysaccharide interactions, the inclusion of plant protein-generated flavors, and some novel techniques to structure plant proteins are discussed. Finally, the attention to nutrition as a compass to navigate the plant protein roadmap is also considered.

Although the focus of this review on plant proteins is in the context of advanced nations, it is important to recognize that over one billion people may suffer from protein deficiency [10]. This problem may be more apparent in South Asia and Africa. We therefore hope that this review will also stimulate scientists to consider how they may develop low-cost alternative protein sources for consumption by people in developing nations.

## 2. Developing Plant Protein Ingredients

### 2.1. Protein Structure and Functionality

Proteins are macromolecules comprising linear polymers of amino acid residues joined by peptide bonds which have various structural, functional and nutritional properties that are useful for the food industry in food formulations [11]. The physicochemical properties of proteins such as surface hydrophobicity, net charge, and the presence of reactive groups of a protein depend on the factors such as the type, number, order, orientation of its amino acids and interactions among them [12]. Additionally, the molecular size, shape of proteins, physicochemical properties and processing conditions determine its functional properties (gelation, solubility, thermal stability, emulsification, foamability) and affects the protein’s interaction with other macro and micro molecules, processing, storage, isolation, utilization and degradation [13,14].

Due to the growing interest in producing plant-based foods, it is important to choose a suitable plant protein with good functionality to mimic animal proteins. Knowledge of the structure and functional properties of animal proteins provides opportunities to emerging plant protein sources, with a focus on the development of plant-derived protein ingredients and their use in formulated foods. In a food system, proteins are the most significant functional component because of their structuring, texturizing, emulsifying, foaming, hydration, and nutritive properties [15]. Modifying or fabricating plant proteins into structural arrangements that give physicochemical and functional properties such as those supplied by animal proteins is a key problem in this field. In this section, a brief overview of some of the most important structural and functional properties of plant and animal-based proteins are discussed.

**Figure 2:**
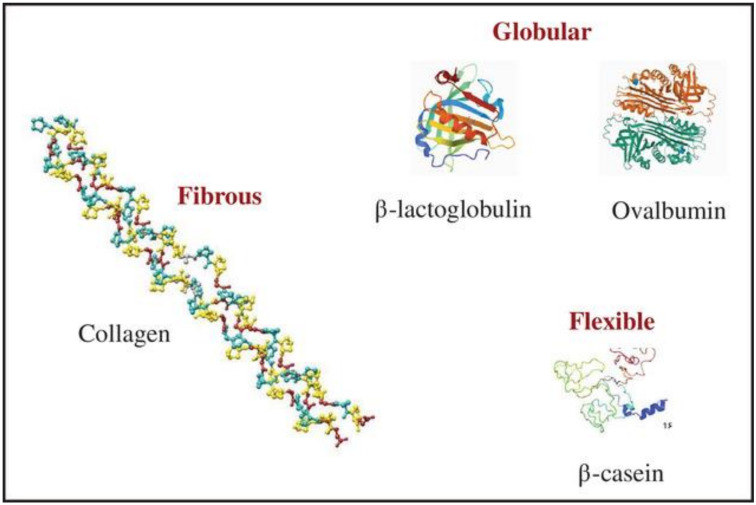
Illustration of proteins obtained from animal sources that differ in structure and functionality. Taken from [4] with permission.

Broadly, based on the structure, proteins can be classified as globular (albumins, globulins, prolamins, glutelins, nucleoproteins, glycoproteins, phosphoproteins, and hemoglobins), fibrous (keratin, myosin fibrin, and collagen), or flexible (casein), depending on their physicochemical properties such as amino acid residue quantity and sequence on the polymer chain (Figure 2). The fibrous structures are generally water-insoluble, whereas various globular structures are soluble in water, acids, and bases [4,16].

#### 2.1.1. Globular Proteins

Plant proteins are mainly made of globular proteins, present as multimers that are covalently linked together, and are classified as albumins (soluble in water), globulins (soluble in dilute salt solutions), prolamins (soluble in aqueous ethanol solutions), and glutelins (soluble in dilute acid/alkaline solutions or insoluble in water) [16]. Albumin and globulins are predominately present in all pulses (>50%) [17] and some pseudocereals (quinoa, and amaranth), whereas prolamins (wheat, maize, barley and rye) and glutelins (wheat) make up 85% of total protein in the cereal [18] and pseudocereal family [16,19]. These globular proteins are made up of polypeptide chains that fold into a densely packed shape due to hydrophobic effects, hydrogen bonding, electrostatic forces, van der Waals forces, and disulfide bonds. The structures of prolamins and glutelins are very similar in terms of the proportion of proline and glutamines and amino acid sequences; however, they differ in molar mass and, intra- and inter-molecular structures [20].

Factors that affect the solubility of globular proteins include (a) differences in molecular weight, (b) molecular flexibility, (c) the presence of hydrophobic, basic and acidic subunits on the protein surface and (d) association and dissociation of subunits [21,22]. For instance, albumins have a greater solubility from neutral to acidic pH than globulins due to their lower molecular weight (10–18 kDa), which enhances structural flexibility and hydrophilicity [23,24,25]. In addition, higher levels of negatively charged amino acids and lower contents of hydrophobic and aromatic amino acids caused the albumin to be highly soluble [26]. Within the class of globulins, 7S globulins have a high solubility (>90%) at pH 3–9 and high ionic strengths (0.5 M), but most 11S globulins have a low solubility (80%) at pH 5–6, except for soy 11S, which has a high solubility (>80%) at pH 3–4 [22,27]. Glutelins and prolamins have low water solubility at neutral pH [28].

Globular proteins act as good emulsifying and foaming agents due to non-polar regions on their surfaces that promote adsorption to oil–water or air–water interfaces. Albumins from pea and chickpea have lower overall emulsifying properties than globulins, due to low molecular flexibility and hydrophobicity [29,30]. On the other hand, albumins from kidney beans have good foamability and emulsification properties due to good solubility, smaller molecular weight, and better molecular flexibility, but they cannot stabilize foams at neutral pH in comparison to globulins [23]. Globulins can form stronger interfacial films, which is likely due to unfolding and interactions between the larger subunits. Additionally, depending on the type of globulin fraction used, foaming and emulsifying characteristics may vary. Individual subunits within both 7S and 11S globulins undergo dissociation, unfolding, and reaggregation in food processing at the right ionic strength and heating conditions, making them more functional in terms of solubility, gelation, emulsification, and foaming [31]. For instance, the differences in the functionality of 7S and 11S globulins are stated as follows: 11S globulins from soybeans and peas are efficient in foam stabilization [32], whereas 7S globulins from soybeans and peas are efficient emulsifiers [33,34,35]. This is due to greater contact with the bigger subunits at the interface. In contrast, in fava beans, 7S globulins have lower emulsification than 11S globulins [27]. The glutelins and prolamins have high molecular weight fractions and low water solubility at neutral pH, which subsequently affects the foamability and emulsification of the proteins. However, native prolamin from wheat is an effective foaming agent, particularly in the alkaline pH range [36].

Globular proteins can also be employed as gelling agents, because when heated, they unfold, exposing non-polar and sulfhydryl groups to the surrounding aqueous phase, causing aggregation via hydrophobic and disulfide bond formation [4]. Gelation occurs when the aggregated proteins arrange into an ordered matrix with a balance of protein–protein and protein–solvent interactions, which are maintained by a balance of attracting and repelling forcible forces [37]. Albumins, globulins, glutelins, and prolamins may all form isotropic (e.g., yogurt-like) or anisotropic (e.g., fibrous structures) gels. When glutelins and prolamins are hydrated, they self-assemble into a gel network, but albumins and globulins require physical, chemical, or enzymatic modifications, which are discussed more in the later sections.

Some examples of animal-based globular proteins used in the food industry are whey (α-lactalbumins, β-lactoglobulins) and albumins (egg white), which are water-soluble and have excellent foaming, emulsifying, and gelling properties [38,39]. Although most plant proteins (soy, pea, potato, mung bean, and rice proteins) are globular [40], they differ from animal globular proteins in terms of molecular characteristics and functional properties. For example, although soy and pea protein have comparable solubility and emulsifying characteristics to egg protein, many plant proteins only denature at higher temperatures (e.g., around 90 °C for soy glycinin compared to ~63–93 °C for egg albumins) [41]. As a result, higher temperatures or longer heating times are often required to achieve the same structure formation and textural attributes as real eggs. In addition, plant globular proteins are subjected to extraction and isolation for food applications. This can cause denaturation and aggregation, affecting the functionality of the plant proteins [41].

#### 2.1.2. Fibrous Proteins

Fibrous or meat proteins have a complex hierarchical construction of fibrous protein bundles embedded inside connective tissue formed of triple helices of collagen, and can be classified as sarcoplasmic, stromal (collagen, elastin), and myofibrillar (myosin, actin, tropomyosin, troponins) [42,43]. Depending on the animal species and muscle kinds, one individual muscle fiber (i.e., a cell) measures 1–40 mm in length and 20–100 mm in diameter [44]. Collagen is derived from animal tissues which are made up of interconnected polypeptide chains. Actin and myosin molecules in muscle tissues are packed into fiber-like structures, which contribute to the textural qualities of foods. There is a long history of creating and manufacturing mock meat products, and a large proportion of research has focused on recreating the fibrous structure from plant proteins. Comparing the fibrous characteristics of animal to plant protein, only glutenin in wheat has cohesive and viscoelastic characteristics, enabling it to form fibrous proteinaceous networks which are widely utilized in alternative meat products. To produce meat like-products from other plant-based proteins is a challenge due to their nature. Texturized vegetable proteins (TVP) have been manufactured in the food industry for a very long-time using extrusion, which helps to recreate a fibrous structure to globular plant proteins. With modification or treatments to plant proteins, fibrous meat-like proteins can be developed, as discussed in the later sections of this review.

#### 2.1.3. Flexible Proteins

Filamentous proteins have flexible and disorderly structures; for instance, casein has a random coil structure, with hydrophobic and hydrophilic patches [45]. Casein forms large colloidal particles with calcium phosphate to form casein micelles [46,47]. Casein has excellent surface-active and stabilizing properties due to the following factors: (a) high proportions of prolyl residues conferring open and flexible conformations; and (b) random coil shapes that include hydrophobic, hydrophilic regions and phosphate groups [46]. Caseins are highly stable at neutral pH but can coagulate with an acid or enzyme. Caseins bind together to form three-dimensional networks that give them their distinct textural qualities which have a significant impact on the production and qualities of cheeses and yogurts. These molecular properties differ significantly from those of globular plant proteins. The caseins’ surfaces feature polar and non-polar areas, making them good emulsifiers. They also include a lot of anionic phosphate groups, which means calcium ions may bind them together, which is crucial for gel formation. Gelatin is a flexible protein made up of collagen, which is a superhelical structure made up of three parallel alpha-chains. When exposed to heat, it melts, but when chilled, it forms helical sections that may crosslink with one another via hydrogen bonding; thus, it has good cold-set gelation characteristics [48].

Simulating the characteristics of gelatin and casein has proven extremely challenging because most known natural plant proteins do not have flexible random-coil structures or micellar structures, respectively. Assembling plant proteins into these superstructures is therefore a key problem in the field. Although certain polysaccharides that could simulate the properties of these flexible proteins exist, more attention could be paid to recreate these flexible structures in plant proteins. This could take shape by introducing random coils in globular plant protein structures or the assembly of various plant proteins to mimic casein micellar structures.

### 2.2. Plant Protein Extraction and Fractionation

To fully realize the potential of plant proteins as described in the previous section, the proteins must be extracted and fractionated intact. This is challenging compared to some animal proteins. For instance, egg white albumin is relatively easy to separate from the egg yolk, and dairy casein is highly resistant to various forms of processing. Historically, alkaline/acid extraction and precipitation has been extensively used to isolate and fractionate proteins. However, early observations show that the alkaline treatment of protein leads to the production of lysinoalanine, a toxic amino acid [49]. Most commercial plant protein isolates available today employ a wet extraction and fractionation technique (alkaline extraction–isoelectric precipitation) that also causes extensive protein denaturation and aggregation, severely affecting functionality because of the harsh conditions used [50]. For example, up to 75% of proteins present in pea protein isolates are insoluble and non-functional, and hence, unutilized [51]. In addition, endogenous phenolic compounds may form complexes with plant proteins, affecting the functional and nutritional properties of the proteins [52]. These considerations therefore necessitate the development of new and novel methods of protein extraction and isolation. The academic community, working together with industry, has acknowledged this challenge, and has started to seek out different strategies based on the wide variety of plant protein sources [6,53]. The following discussions present some of the considerations. Wet protein extraction typically involves tissue disruption and protein solubilization, following which the protein ingredients are generally obtained by precipitation and concentration [16]. It is also timely to mention that commercial plant protein ingredients are derived as isolates (>80% protein concentration), concentrates (50–60% protein concentration) and flours (~20% protein concentration).

#### 2.2.1. Plant Protein Extraction

The overview of plant protein extraction was well covered in a recent review [54]. Depending on the source material, different approaches are used. For example, mechanical dehulling and milling are used to disrupt plant seeds, whereas high-shear blending is better suited for grasses. Depending on the plant matrix, various solvents such as acids, bases, salts, organic solvents, and more recently, subcritical water [55], are used to defat and/or solubilize the proteins. Different processing conditions are also used to optimize the extraction process. However, the use of acids and bases in certain cases may cause protein damage, whereas organic solvents are less environmentally friendly. As mentioned above, the alkaline treatment of proteins can also lead to the production of lysinoalanine. Therefore, new methods of protein extraction are needed. A promising alternative is to use food-grade deep eutectic solvents (DES). As a green and mild solvent, DES has shown promise in extracting various food components such as phenolic compounds and sugars [56]. There are also recent proof-of-concept studies using DES to extract oilseed cake protein [57] and oat protein [58]. Although encouraging, more work is needed to design other food-grade DES solvents, to improve our understanding of the extraction chemistry, to optimize the process, and to increase the extracted protein content (currently ~50%).

To further improve the extraction efficiency, additional physical and/or enzymatic techniques can be used. These include ultrasound, pulsed electric field, microwave, high-pressure, pectinases, carbohydrases and proteases [54]. The general principle is similar: to weaken or disrupt the plant cellular matrix, notably, the disruption of the polyphenol–protein and fiber–protein complexes, so the extraction solvent can more effectively penetrate. Additionally, some techniques such as supercritical fluid extraction can further remove lipids and polyphenols bound to proteins [59]. However, besides cost, an important deliberation is whether these processes will also disrupt the protein structures, rendering the extracted proteins less functional. In that respect, nonthermal processing methods may be favored. Another important but less explored consideration is if these processes could also alter the solvent extraction properties. For example, high-pressure processing was found to reduce the in situ pH values of liquid foods during processing [60]. This could be exploited to improve acidic extraction methods. Thus, a careful balance of these factors can promote the affordable, sustainable, and efficient extraction of intact and functional plant proteins.

#### 2.2.2. Plant Protein Fractionation

After extraction, the proteins are purified and separated from the extraction solvent. For alkaline-extracted proteins, isoelectric precipitation is the most common method to obtain high-concentration protein isolates. This process takes advantage of the proteins aggregating at their isoelectric points, which can be separated by decanting and centrifugation. As mentioned earlier, this process has detrimental effects on protein structure and functionality, and typically only the globulin fraction is obtained. A gentler alternative to isoelectric precipitation is ultrafiltration and/or diafiltration, whereby specific protein fractions, including albumins, can be isolated by molecular weight without sacrificing protein concentration and functionality [61]. However, membrane filtration is susceptible to fouling, which reduces the isolation efficiency [62]. To reduce fouling, membrane scientists have been actively researching novel membrane materials and strategies [63,64]. Food scientists will need to work closely with engineers to ensure that these membranes are compatible and safe for food applications. Another mild option is to use salt extraction. This method capitalizes on the salting-in effects on proteins, and does not require extreme pH or elevated temperature conditions [65]. In addition to ultrafiltration, methods to isolate the salt-extracted proteins involve desalting steps such as micellar precipitation and dialysis. However, these salt extraction and desalting processes only work for globulins, require a large amount of water, and may be more energy-intensive. To determine the best approach, a recent trend is to perform direct comparisons of the various extraction and fractionation methods. It was found that different methods can modulate the protein composition, structure, and functionality [66,67]. More work is needed to integrate these findings into commercial processes. Some other new ideas involve using fermentation to assist protein extraction [68] or redesigning the wet fractionation process to be milder [69]. Some authors even suggest to selectively extract the albumin fraction (under acidic conditions) to obtain a functional protein isolate while using the residual proteins for feed [70]. The Flory–Huggins solution theory [71] is often used as a theoretical basis to design better protein extraction methods [16]. By making opposite considerations to protein solvation, one could use the theory to expand the protein isolation toolbox beyond isoelectric and micellar precipitation. These techniques should encourage protein–protein, protein–other molecules, and solvent–solvent interactions, while disrupting protein–solvent interactions. At the same time, the techniques must compensate for the loss of entropy from protein–solvent separation. A potential example is supercritical antisolvent fractionation [72].

In our opinion, a promising commercial isolation technique to obtain functional plant proteins is membrane filtration. To improve cost efficiency, one could take a leaf from the dairy industry whereby whey protein isolate is produced in bulk using ultrafiltration [73]. Some important factors for its success include (1) the use of previously low-value side-streams as feed (i.e., sweet whey, a by-product of cheese making), (2) economic scalability, and (3) production of high-value products (i.e., whey protein isolate for sports nutrition). Some potential equivalent feed materials include spent grains, coffee grounds and oilseed press cakes [74]. These materials have a portion of non-protein fraction already removed (e.g., starch in brewer’s spent grain) and are usually discarded [75], making them promising feed materials for protein isolation. Readers are referred to this recent review for more in-depth discussion [76]. Apart from traditional food operations such as tofu and soy sauce making, it is also anticipated that the plant-based movement will generate new product categories and unit operations from which new side-streams can arise.

Wet extraction and fractionation are typically energy- and water-intensive processes and also generate proteinaceous effluents [50]. To improve sustainability, dry fractionation methods such as air classification and electrostatic separation are currently explored [77,78,79]. These techniques exploit the density and particle size differences between protein and other components to obtain protein-rich concentrates. In addition to being more energy efficient and solvent-free, the proteins are obtained structurally intact, and hence, functional. However, more work is needed to improve protein concentration. Present research includes studying hybrid or combined air classification with electrostatic separation processes.

Most commercial protein ingredients are made up of various protein fractions, such as albumins, globulins, glutelins and prolamins. Even most legume protein isolates rich in globulin contain various globulin subfractions, such as legumin, vicilin and convicilin in pea [65]. These fractions each have different techno-functionalities which affect the overall protein ingredient performance based on its proportion in the ingredient. For example, it was discovered that pea albumins form stronger heat-set gels than pea globulins [80]. Within pea globulin subfractions, legumin is detrimental to acid gel formation [81] and has poorer emulsifying properties than vicilin [34]. The cysteine-rich legumin is, however, useful for nanoparticle development for drug delivery applications [82]. It would therefore be useful to separate different plant protein fractions for specific applications, or to obtain fraction-enriched protein ingredients that can be tuned having different fraction proportions for targeted techno-functionalities. Although separating albumins from globulins is relatively easy, it is difficult to separate the subfractions because they have similar isoelectric points, solubility, and molecular weights. Protein subfractions can be separated by chromatographic techniques, but the separation efficiency is too low for bulk applications. New ideas are needed, therefore, to improve the separation efficiency of plant protein subfractions.

### 2.3. Modification of Plant Proteins

Plant proteins typically have relatively poor nutritional and functional properties compared to animal proteins, including dairy. This is exacerbated due to the harsh commercial protein isolation conditions, as described in the previous section, which cause some protein denaturation, aggregation, and loss in functionality. Due to aggregation, these non-functional proteins are usually insoluble and may have deleterious effects on the overall performance of the protein ingredient. These non-functional proteins may be separated by simple centrifugation [83]. This enables plant protein ingredient suppliers to have separate lines of plant protein ingredients for optimized applications (e.g., NUTRALYS S85 soluble pea protein and B85 insoluble pea protein, Roquette, France). On the other hand, dry fractionated plant protein concentrates have structurally intact proteins and therefore better functionality. However, the lower protein content and presence of other impurities pose challenges to product formulation and high protein claims, limiting their applications.

To improve functionality, the plant proteins may be modified. Two recent reviews comprehensively cover this area [8,9]. Akharume and coworkers elegantly describe the general strategy involving physical, chemical, and biological perturbations to the protein structure and conformation to improve or create new functionalities [8]. We note the active research in emerging technologies to modify proteins such as cold plasma [84] and various forms of electromagnetic waves/fields [85]; this section covers a few innovative physical, chemical, and biological modifications that could easily be adopted by the food industry. An interesting research direction is the combination of these technologies on plant protein structures, and some examples will be discussed.

We would also like to introduce another paradigm in protein modification: the transformation of denatured and/or aggregated non-functional plant proteins into functional plant proteins. Until we adopt better protein isolation techniques, commercial plant protein isolates contain a large fraction of non-functional proteins. Therefore, functionalizing these “inert” plant proteins will unlock greater value for plant protein ingredients. Native and non-functional proteins are in very different conformational states; therefore, the approaches needed to modify each of them are likely to be different. For instance, techniques to improve foaming and emulsifying behavior by protein denaturation will not likely influence the already denatured non-functional proteins. The modification objectives for native and denatured proteins are anticipated to be different; therefore, it is better to separate the non-functional proteins from the native proteins. Some methods to transform non-functional proteins include thermal treatment [86] and micro-fluidization [83,87].

#### 2.3.1. Physical Modifications

##### High-Pressure Processing

**Figure 3:**
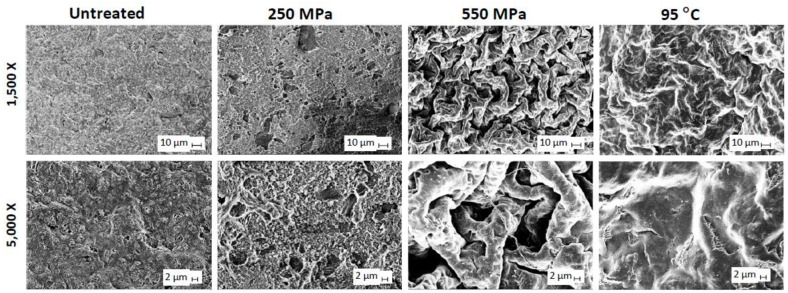
Scanning electron micrographs of untreated, pressure-treated, and heat-treated 24 g/100 g pea protein concentrate solutions. A greater extent of aggregation and network formation is observed after higher pressure treatments. Adapted from [94] with permission.

High-pressure processing (HPP) is a nonthermal processing method where food is subjected to high hydrostatic pressures (200–600 MPa) and held for a certain amount of time (3–5 min). All food components experience the same pressure quasi-instantaneously. Currently, the primary use of HPP is cold pasteurization and shelf-life extension, but HPP has recently been explored for food structure engineering [88]. This is possible through the pressure-induced structure modifications of macromolecules, such as protein denaturation and starch gelatinization. HPP has little effect on the protein primary structure, but can disrupt non-covalent interactions. The extent of protein structure destabilization depends on the amount of pressure applied. The oligomeric subunits in globular proteins can be dissociated under moderate pressure (50–200 MPa) and are reversible [89]. Pressures above 300 MPa, however, cause irreversible denaturation and aggregation and may lead to the conversion of one secondary structure to another [90]. The pressure-induced protein structure changes can alter its functional properties such as increased solubility, water holding capacity, emulsifying and foaming propensity, and the formation of gels [91]. A variety of protein gel structures can be created by controlling pressure and which differ from heat-set gels (Figure 3). Readers are referred to recent reviews on plant proteins [92,93] for more details.

##### High-Pressure Homogenization

High-pressure homogenization (HPH) is a technology that produces a homogenous size distribution of particles suspended in a liquid by forcing through a narrow orifice or valve [95]. HPH can be used to modify plant proteins by improving their functionality. The mechanical forces from HPH lead to the cavitation and fragmentation of macromolecules by reducing the particle size of the plant proteins. For instance, Saricaoglu et al. reported that the increase in homogenization pressure progressively decreased the volume-weighted mean value of hazelnut proteins [96]. The particle size of the control was found to be highest (203.92 µm), whereas the sample treated at 150 MPa was the lowest (58.91 µm). Saricaoglu reported the same trend on lentil proteins using HPH [97]. Porto et al. mentioned that the maximum pressure of HPH at which the smallest particles can be obtained depends on the type of protein and solvent used [98]. However, beyond these two factors, there may be an increase in particle size because of protein denaturation due to mechanical forces in the pressure drop and elevated temperature reached in the homogenizer valve.

The solubility of the protein increased, as well as higher emulsifying and foaming properties observed because of decreased particle sizes. For example, Saricaoglu et al. reported an increase in hazelnut protein and lentil protein solubility when the pressure reached 100 MPa [96,97]. However, an “over-processing” effect was observed at 150 MPa homogenization pressure, where the protein solubility decreased. The emulsifying activity index (EAI) and emulsion stability index (ESI) of hazelnut and lentil protein increased as the pressure of HPH increased from 0 to 100 MPa, but then decreased when the pressure reached 150 MPa [96,97]. The authors explained that pressure led to the partial dissociation and unfolding of proteins, which resulted in a higher hydrophobic and hydrophilic interaction of protein by improving the efficiency to form interfacial layers. The foaming capacity and foaming stability increased with increasing pressure, whereas a significant decrease in foaming properties was observed for hazelnut protein and lentil protein at 150 MPa [96,97]. The increasing foaming properties could be attributed to smaller protein particles occurring, which led to the fast movement of proteins towards the air–water interface, whereas the decreased foaming properties were due to reduced intermolecular interactions and the further unfolding of proteins.

HPH also leads to a change in protein structure. For instance, Yang et al. reported an increase in α-helix and β-turns, and a decrease in β-sheets in faba bean protein was observed after HPH at ~206.8 MPa [99]. HPH caused a particular impact on hydrogen bonds, resulting in some changes in the secondary structure of faba bean proteins. Guo et al. also reported the same trend on kidney bean protein [100]. The authors also reported that the components of the intramolecular β-sheet first decreased and then increased. In contrast, the components of β1, β2, and random coil first increased and decreased with increasing pressure. This could be due to the high pressure and high shear forces of HPH at a lower pressure disrupting the soluble aggregate structure by weakening the protein–protein interactions, reflected as an increase in β1, β2 and random coil [100]. Overall, HPH demonstrated a certain level of protein conformation change (e.g., tertiary and quaternary structure levels) when subjected to high pressure.

##### Extrusion

Extrusion is a process in which a material is forced to flow, under a variety of conditions of mixing, heating and shearing, through a die designed to form or puff-dry ingredients [101]. The high-temperature short-time (HTST) process conditions of extrusion has an impact on protein functionalities such as solubility, texture, emulsifying and gelation properties [8]. Extrusion processing is particularly used for texturizing plant proteins (e.g., soy protein, pea protein and wheat gluten) to generate high-moisture meat analogues [102,103]. The macromolecules in the proteins lose their native and organized structure when undergoing thermal and mechanical stresses, as a result of heating of the barrel and shearing of the screws during extrusion [104].

The high temperature during extrusion can cause protein unfolding due to the breakage of hydrogen bonds [105]. The intramolecular disulfide bonds are broken down with a further increase in temperature, leading to the formation of new intermolecular bonds (e.g., hydrogen, hydrophobic or disulfide), resulting in protein aggregates [106]. The high pressure and temperature during extrusion can also destroy anti-nutrients and improve the digestibility of plant proteins by increasing the availability of their amino acids [9]. Nosworthy et al. reported that extruded chickpea flour had the highest amino acid scores, in vivo digestibility and protein digestibility-corrected amino acid score (PDCAAS) when compared to cooked and baked chickpea flour [107]. Omosebi et al. reported higher in vitro protein digestibility on extruded soybean protein concentrates than uncooked samples [108]. However, insufficient explanations and justifications were presented as to why extrusion was the optimal method for the modification of chickpea flour with the highest protein quality, or why processing conditions during extrusion contributed to a higher protein digestibility by the authors.

Extrusion can also be used as a pre-treatment for other protein modification methods, such as ultrafiltration and enzymatic hydrolysis. Dehulled black beans were processed through extrusion before being extracted and concentrated by ultrafiltration, followed by spray-drying [109]. Peanut and pea protein isolates were also modified with extrusion as pre-treatment, followed by proteolysis using papain [110,111].

##### Sonication

Sonication or ultrasound processing is one of the emerging technologies that has shown potential in the food industry [112]. This process applies sound energy at extremely high frequencies (>20 kHz) to break down large particles in a solution through physical vibration, using either an ultrasound probe (e.g., sonicator) or an ultrasound bath [113,114,115]. A cavitation effect is generated where the vibrations create microscopic gas bubbles in the solution, and pockets of spaces wedged between molecules are formed and collapsed to send shock waves into the surrounding area [114,116]. Sonication can also modify protein functionality through localized hydrodynamic shearing of the native protein particles, and by the thermal degradation of protein molecules through heating [116].

Arzeni et al. reported that protein aggregates were violently agitated and collided during sonication, resulting in smaller broken particles and wider size distribution [117]. This was also reported by Xiong et al. on sonicating 5% *w*/*v* pea protein isolate solutions at different amplitude levels of 30, 60 and 90% for 30 min at 20 kHz [118]. In addition, shearing from sonication alters the size of protein particles and their distribution [116]. The size of protein aggregates is reduced due to the disruption of non-covalent interactions, increasing its solubility [9]. This was reported in the sonication of both 1% *w*/*w* wheat and soy protein isolate solutions at a frequency of 20 kHz and amplitude of 95% for 2 min, and also for 0.5% *w*/*v* walnut protein isolate solutions at sonication powers of 200, 400 or 600 W for either 15 or 30 min [115,119]. However, in some cases, larger aggregates are formed after sonication due to low power and long (>20 min) or intense treatment [116]. For instance, Jin et al. reported that the particle size of buckwheat protein isolate increased significantly (fivefold) when sonicated at 100% amplitude for 10 min [120]. The authors explained that the increase in particle sizes might be due to the disruption of protein microstructure by sonication, resulting in swelling of the protein particles in water, or protein self-assembly due to hydrophobic interaction between its unfolded regions [120].

Sonication can induce structural changes due to the disruption of non-covalent bonds. The process can destroy the secondary structure and partially denature the tertiary and quaternary structure of proteins without any significant changes in their primary structure [121]. The reduction in particle size for pea protein isolate after sonication led to the decrease in intermolecular association, resulting in more sulfhydryl (SH) groups and hydrophobic regions to be exposed [118]. Jin et al. also reported an increase in the reactive and total SH contents and decreased disulfide (SS) bond, as sonication exposed the SH buried inside buckwheat protein molecules, and SS bonds were broken down because of the physical and chemical effects of cavitation [120]. The authors also reported that sonication increased the digestibility of buckwheat proteins because of changes in the contents of secondary structures, resulting from cavitation-induced physical forces and free radicals. Zhu et al. reported that increasing the intensity and duration of sonication might have disrupted certain types of hydrogen bonds [119]. Some of the secondary structures of walnut protein isolate, such as α-helix, were converted into β-sheet, β-turns, and random coil.

#### 2.3.2. Chemical Modifications

Chemical modifications include reactions with chemical agents and modification by pH alteration [122]. Derivatization is a process of chemical modification in which the reactive side chains in the proteins’ main structure are chemically altered to affect their physicochemical characteristics and increase their functioning. Derivatization occurs mostly at amino, carboxyl, disulfide, imidazole, indole, phenolic, sulfhydryl, thioether, and guanidine functional groups of proteins. The nucleophilicity of the amino and sulfhydryl groups makes them reactive. As a result, processes including acylation, phosphorylation, esterification, and deamination have been utilized to give food proteins better functional characteristics. The application of these techniques in the food sector is severely limited because of the use of many hazardous chemicals, and subsequently, the unknown food consumption credibility of the modified proteins or its by-products. Additionally, for modifying proteins, more convenient and economical treatments should be utilized. Thus, in this study, glycation and pH shifting will be discussed; they are two promising methods for enhancing the techno-functional properties of proteins. Glycation is the most desired for food applications because it does not need the use of hazardous chemicals and produces no by-products. As a result, this technique may be a suitable chemical modification strategy for plant-based proteins in terms of consumer preferences, clean labels, and commercialization.

##### Glycation

Glycation is a common food-grade reaction which is widely used to improve protein functionalities because it is safe, and it usually does not require exogenous chemicals. Glycation can be achieved chemically through Maillard reactions or it can be obtained by cross-linking enzymes such as transglutaminase or laccase [9]. The latter is discussed in the enzymatic modification section. The Maillard or non-enzymatic browning reaction is a sequence of non-enzymatic processes involving the conjugation of free amino groups of a protein and the carbonyl groups of reducing carbohydrates which alter free or protein-bound amino acids in the presence of reducing sugars [123,124]. The Maillard reaction is preferable to other chemical modification methods because it is a natural and spontaneously occurring process in food that does not require extra chemicals [125]. During the glycation of proteins, temperature, time, concentration of the reactants, pH, and water activity should be controlled to influence the yield, quality, and techno-functionality of glycated proteins, and to limit color and off-flavor formation to a minimum [126]. Traditionally, glycated plant proteins are produced by two common methods: dry-state or wet-state heating methods [124]. Traditional dry- and wet-state heating methods are either excessively expensive or inefficient for commercial use; therefore, innovative ways to form protein–carbohydrate conjugates have recently been devised [127,128]. The use of ultrasonication, pulsed-electric fields, or irradiation to the protein–carbohydrate dispersion to produce high temperatures, and in certain circumstances, accelerate protein unfolding, has been shown to have a favorable influence on the glycation process in a wet state. Other approaches such as high-pressure pre-treatment, extrusion, and electrospinning can produce glycated proteins. Although there is a rise in new innovative technologies to glycate the plant proteins, scaling up the manufacturing process of modifying the plant proteins through glycation are difficult due to the cost. Therefore, efforts should be made to create an industrially viable technique for producing glycated plant proteins. The need for a more sustainable food production system comprising the inclusion of more highly functional plant-based components has increased the requirement for an economically effective manufacturing technique [129].

Several studies have discussed this in-depth, but this section focuses on only a few examples of the glycation-induced structural modifications in several plant-based proteins, resulting in enhanced functionality. For instance, after dry-state glycation, sesame protein had higher solubility and emulsifying ability at a wide range of studied pH, due to secondary structural changes [130]. The foaming and emulsifying properties of soy protein isolate improved after wet-heating glycation with glucose [131]. An improved foaming ability was also reported for soy protein isolate after conjugation with lentinan via ultrasonic-assisted Maillard reaction, together with solubility, emulsifying ability and thermal stability [132]. Glycation was used as an approach for a reduction in the beany flavor and improve flavor profiles in pea protein conjugated by Arabic gum [133]. This can promote the application of plant-based proteins as ingredients in food formulations without any unfavorable effect on their organoleptic properties. Glycation of soy protein isolate with lentinan by wet heating enhanced its foaming capacity and foam stability [132]. Further studies demonstrate a positive impact of glycation on the foaming properties of gluten–fructose conjugates [134] and fava bean protein–maltodextrin conjugates [135]. Although there has been much research on glycated plant proteins characteristics and functional behavior, the relationship between the structure of glycated proteins and its functionality in food is not clearly established. Therefore, designing a process to glycate the protein subsequently to alter the functionality of the plant proteins becomes difficult. More research is needed to fully understand the benefits and drawbacks of glycated plant proteins in various food systems.

##### pH Shifting

Acidic or alkaline treatments can cause changes in the structural and functional characteristics of proteins because the pH of the liquid matrix in which proteins are dissolved is one of the major effective variables on their structure. During pH-shifting, the extreme acid–base environment results in protein conformational change, commonly known as a “molten globule” structure, which improves the emulsification properties of globular proteins [136,137,138,139]. Proteins are denatured and unfolded at basic pH levels, revealing sulfhydryl and hydrophobic regions in their structure and allowing novel protein interactions to emerge [140]. Chemical additives, such as NaOH and NH_4_OH, can readily be used to create alkaline conditions [141,142]. Hydrochloric acid is commonly used to create acidic conditions [9].

Due to the structural alteration, pea protein following pH-shifting treatment under alkaline conditions demonstrated better oxidative stability in oil-in-water emulsions than native pea protein. Changing the pH of wheat gluten to basic levels using NaOH resulted in changes in their molecular and secondary structure, as well as improved extensibility and tensile properties of the resultant films [143]. They promote protein reactivity by increasing its unfolding; thus, pH-shifting treatments can also be employed as a pre-treatment for other modification techniques. The introduction of the pH-shifting method is of great significance to improve the gel properties of peanut protein isolate. It was found that the breaking force and water-holding capacity of the pea protein gel improved significantly due to the reduced particle size, enhanced solubility, free sulfhydryl group concentration, and surface hydrophobicity when the peanut protein isolate was subjected to pH shifting [144]. In another study, pH shifting seemed to enhance the protein solubility in soy; however, the effect of pH shifting was low when the ionic strength was high and the pH was between 4 and 7 [145]. This behavior limits the use of pH shifting in protein isolates that will be used in food applications with low pH or high salt content.

Therefore, for plant-based proteins, most studies have employed pH shifting in conjunction with ultrasonic treatment. Soy protein has improved functionality such as enhanced solubility, surface hydrophobicity and emulsifying ability with the combination of pH shifting and ultrasonication [146]. In another study, soy protein had improved solubility, surface hydrophobicity, antioxidant activity, rheological and emulsifying properties through the combination of pH shifting and mano-thermo-sonication [142]. Rice protein had improved solubility, emulsifying, and foaming characteristics with pH shifting and ultrasonic treatment [147]. A study showed the effects of acidic and alkali treatments with ultrasonication on pea protein. It found that the alkali treatments improved protein solubility and functional characteristics more than the acidic treatments. Additionally, surface hydrophobicity and emulsifying characteristics of pea protein increased following pH shifting and ultrasonic combination treatment [148]. By addressing their poor solubility problem and improving their techno-functionality, the combined treatment of pH shifting and sonication was also used to extend the application of plant-based proteins for the encapsulation of bioactive compounds. Most importantly, ultrasound can inhibit the formation of lysinoalanine during pH shifting [149].

#### 2.3.3. Biological Modifications

Protein modification using enzymatic methods can be classified into enzymatic hydrolysis or enzymatic cross-linking. Enzymatic hydrolysis is carried out by breaking protein peptide bonds to improve its biological and nutritional value, creating hydrolysates of high-added value [150]. On the other hand, enzymatic cross-linking is achieved by forming covalent bonds using transglutaminase, by the catalyzing acyl transfer reaction between the γ-carboxamide group of protein-bound glutamine and ε-amino group of lysine [9].

Transglutaminase and other oxidative enzymes are used to induce the cross-linking of proteins, to improve the textural properties of the proteins by building up the polypeptides into stronger structures [8]. To date, transglutaminase is the only commercially available food-grade cross-linking enzyme, and it has been reported to improve the functional and structural properties of proteins. Sun and Arntfield reported a reduction in minimum gelation concentration for pea protein isolate from 5.5% to 3% *w*/*v* when transglutaminase was added at 10 U/g [151]. The addition of transglutaminase promoted cross-linking among protein molecules and increased the gelation ability. Nivala et al. reported the decreased soluble protein of faba bean isolate from 77% to 60% when increasing transglutaminase dosage from 10 to 1000 nkat/g [152]. The authors also reported that the vicilin and α-legumin of faba bean isolate were prone to form cross-links to higher oligomers, especially at the highest dosage of transglutaminase.

Enzymatic hydrolysis is able to modify structural properties; for instance, the structure of rice bran protein was unfolded, which exposed the buried hydrophobic groups to the outer surface of the molecule [153]. Zang et al. also reported an increase in α-helices and random coil contents, and a decrease in β-sheet content when compared with native rice bran protein. Enzymatic hydrolysis caused the secondary structure of rice bran protein to expand and become more flexible, which is useful for improving its emulsification properties. Zang et al. also reported that moderate hydrolysis (3% DH, degree of hydrolysis) increased the emulsifying properties of emulsions stabilized by rice bean protein, because the increase in droplet diameter remained small for the emulsion. Beaubier et al. reported a higher emulsifying capacity of sunflower protein hydrolysates at 8% DH than 6% DH using Alcalase^®^; however, no significant difference was observed for the emulsifying stability, foaming capacity and stability [154].

Enzymatic hydrolysis breaks down the peptide bonds in the protein; therefore, the molecular weight of the protein is also reduced. Klost and Drusch reported that convicilin (~70 kDa), legumin (~60 kDa) and vicilin (~50 kDa) of pea protein hydrolysate were no longer detected in the SDS-PAGE profile after tryptic hydrolysis [155], although a variety of smaller peptides, including one prominent peak at ~47 kDa, were observed. The increased functionality such as solubility, emulsifying and foaming properties of hydrolyzed plant proteins has been attributed to size reduction. For instance, Klost and Drusch reported a significant increase in solubility for pea protein hydrolysate from 30% (0% DH) to ~60% (4% DH) at pH 5 [155]. This was attributed to the increased amount of terminal carboxyl- and amino groups. Zang et al. explained that the release of soluble peptides from insoluble aggregates or precipitates, and an increased number of exposed ionizable amino and carboxyl groups, were the reasons behind the increased protein solubility and DH [153]. Schlegel et al. reported that lupin protein hydrolysates showed a significant increase in foaming activity, as compared to the native sample [156]. The increase in foaming activity was a result of the change in protein structure, which exposed the hydrophilic and polar groups to interactions with an aqueous environment.

Enzymatic hydrolysis can also be used as a pre- or post-treatment for other protein modification methods, such as ultrafiltration and high-pressure processing, respectively. Aguilar et al. produced black bean protein hydrolysates using Alcalase^®^, Flavourzyme^®^ and Neutrase^®^ (Novozymes, Bagsværd, Denmark). The hydrolysates were fractionated using ultrafiltration with molecular weight cut-off membranes of 30, 10 and 3 kDa [157]. Guan et al. hydrolyzed soy protein isolate by Corolase^®^ (AB Enzymes, Darmstadt, Germany). at an enzyme-to-substrate ratio of 3:100 under a high hydrostatic pressure of 80–300 MPa [158]. The high-pressure treatment unfolded the protein structure, exposing the cleavage sites on which the enzymes could act. The digestibility, allergenicity and antioxidant activity of plant proteins can be improved by enzymatic hydrolysis. For instance, Venuste et al. reported an increase in the in vitro protein digestibility of pumpkin meal from 71.32% to 77.96%, after hydrolyzation by Alcalase^®^ [159]. Wang et al. reported that the allergenic proteins of soybean meal (e.g., α’, α and β-subunits of β-conglycinin) were greatly reduced upon hydrolysis using Alcalase^®^ rather than Trypsin [160]. Zhao and Xiong reported that soy protein hydrolysate at 5% DH improved the emulsion oxidative stability, due to its strong radical scavenging activity [161].

Interestingly, a compound known as plastein was found to be a product of protease-induced aggregation of protein hydrolysates, or from peptide-rich mixtures. These aggregates were initially believed to be formed through the reformation of peptide linkages [162] and were similar to acid-denatured proteins. However, the exact role of proteases in the aggregation process remains unclear. Over the years, many other mechanisms have been proposed for the formation of this compound, such as through non-covalent bonds from peptide interactions [163,164,165,166]. Plastein was identified to be a vehicle for the debittering of protein hydrolysates in foods, but upscaling was faced with many challenges, mainly with yield and production costs [167]. Hence, future work can explore further understandings of the mechanism of plastein formation, which may present solutions in its upscaling for food applications.

## 3. Creating Future Foods Using Plant Proteins

The previous sections have described the gaps in plant protein ingredient science and technology. However, we usually eat foods, and not individual ingredients. After obtaining highly functional plant proteins, the challenge is to transform these ingredients into delicious and nutritious foods. The following sections describe some important factors: the role of protein–polysaccharide interactions, the ability to structure plant proteins into fibers and gels, the inclusion of flavors derived from plant proteins, and nutrition to guide the development of plant-based foods.

### 3.1. Protein–Polysaccharide Interactions

Most foods are a complex mixture of various components. In addition to proteins, polysaccharides make up the predominant component in most plant-based ingredients. Polysaccharides are sugar polymers linked by glycosidic bonds and include a vast family such as starch, cellulose, pectins, agars, carrageenans, alginates and gums [168]. By capitalizing on the natural polysaccharides found in many plant protein sources, less-refined plant ingredients could be utilized, because polysaccharides also form the major building blocks in food products as structuring and stabilizing agents through their thickening, emulsifying, and gelling properties [169]. When used in combination with proteins, their functionality can be further expanded through mutual biopolymer interactions [170]. Hence, there is great interest in understanding and controlling protein–polysaccharide interactions to design plant-based foods such as plant-based milks, ice cream and pudding.

From a search of the literature, a total of 49 articles relating to plant proteins with polysaccharides were found published between 1990 and 2021, with the majority (30 articles) published in the last five years. Although this is reflective of the present emphasis on plant protein research, the number of studies is still a small fraction of the entire plant protein research field. Hence, there is a great opportunity to explore deeper into this area. In addition to advancing basic knowledge, polysaccharides could help overcome some functional shortcomings of plant proteins, with the potential to replace animal proteins [171,172]. The following sections summarize the key movements in this area.

The solubility of plant proteins is relatively low; therefore, the addition of polysaccharides has been employed to improve overall biopolymer solubility, and this is often coupled with a processing or modification step. Some examples include simple complexation [173,174], sonication [141,175] and conjugation [130,176,177]. Most notably, some authors report that the biopolymer solubility improved close to the protein isoelectric point [141] with minimum protein solubility shifting towards more acidic regions [173]. This is likely due to a change in net biopolymer surface charges upon complexation and modification. The shift in the apparent biopolymer isoelectric point will be useful for developing acidic beverages with high protein content, to reduce the precipitation of plant proteins. This strategy deserves further examination such as including other types of processing methods.

In addition to solubility, polysaccharides also improve the viscosity [178,179], foaming [174,180,181,182], emulsifying [183,184,185,186,187,188,189,190,191] and gelling [94,192,193,194,195,196,197] properties of plant proteins. Although the alteration of biopolymer interfacial properties would have obvious effects on foaming and emulsifying properties, an interesting approach is to leverage on the poor solubility of plant proteins, to create insoluble plant protein–polysaccharide particles as Pickering emulsion stabilizers [187,188]. Furthermore, it is important to note that processing plays an important role. For example, the thermal treatment of plant protein–starch mixtures led to a mixed protein–starch gel network [198,199], whereas high-pressure processing resulted in starch granules remaining intact and ungelatinized, acting as a filler in the pressure-induced protein gel matrix [196]. High-pressure processing can also kinetically arrest protein–polysaccharide phase separation through pressure-induced gelation, because the transmission of hydrostatic pressure is quasi-instantaneous compared to thermal gradients found in conventional heat processing. This suggests promising directions to explore further.

Polysaccharides do not always improve plant protein performance [200]. In some cases, it may even worsen their properties (e.g., reducing solubility and foaming capacity due to the formation of insoluble electrostatic complexes [201]). More work is needed to understand how these situations occur. In addition to environmental factors such as pH, biopolymer concentrations and ratio, and ionic strength of the system, some intrinsic factors influencing protein–polysaccharide interactions include the shape of the plant proteins. For example, globulins are spherical and highly charged compared to the more extended and charge-diffused gliadins; these differences can affect the interactions and phase separation with various polysaccharides [202]. Another important deliberation is the natural state of the protein. As discussed previously, most commercially available plant protein isolates tend to be largely denatured, and it has been shown to affect the interactions and resultant properties with polysaccharides [203]. To round off this section, other active research areas include complex coacervation [204,205,206,207,208,209,210] and applications in encapsulation [211,212,213,214].

### 3.2. Structuring Plant Proteins

#### 3.2.1. Formation of Fibrous Structures

Replicating the characteristics of muscle tissue comprising muscle fibers, connective tissue, and adipose tissue arranged into complicated hierarchical systems with viscoelastic textural characteristics has proven challenging. The physicochemical and sensory characteristics of traditional meats are largely determined by the structural arrangement of these tissues. During cooking, the thermally unfolded proteins are cross-linked into a continuous gel structure [40]. The firmness and elasticity of the gel are due to the increased hydrogen bonding during cooling. These structural components are responsible for adhesiveness, viscoelasticity and juiciness [215]. In the plant protein industry, the widely used plant proteins are soy, pea and wheat owing to their availability, cost, and processing functionality. Plant proteins are globular, which does not allow for the formation of a meat-like fibrous texture. Having said that, physical modifications such as extrusion and fiber spinning are required to convert native globules into fibers. Soy (140–375 kDa) and pea (150–400 kDa) have a high molecular weight and high surface hydrophobicity, which undergo structural alterations to form polymers during physical modification, and can therefore be texturized to produce products with textural qualities comparable to meat. Mung bean and chickpea isolates presented good gelling properties and formed heterogeneous and porous networks when mung bean flour was extruded to make meat analogue products [216]. Wheat has a significant amount of gluten and possesses special film-forming characteristics that result in meat-like fibers [217]. Wheat gluten is also used in combination with legume proteins, which contributes to meat-like chewiness [102]. Due to the physicochemical differences between animal and plant proteins, it is difficult to reproduce the complex structure of meat fibers, i.e., highly organized fine texture and the water-binding capacity of meat to give plant-based alternatives a meat-like mouthfeel. A potential method to create fibrous structures is thermomechanical processing.

**Figure 4:**
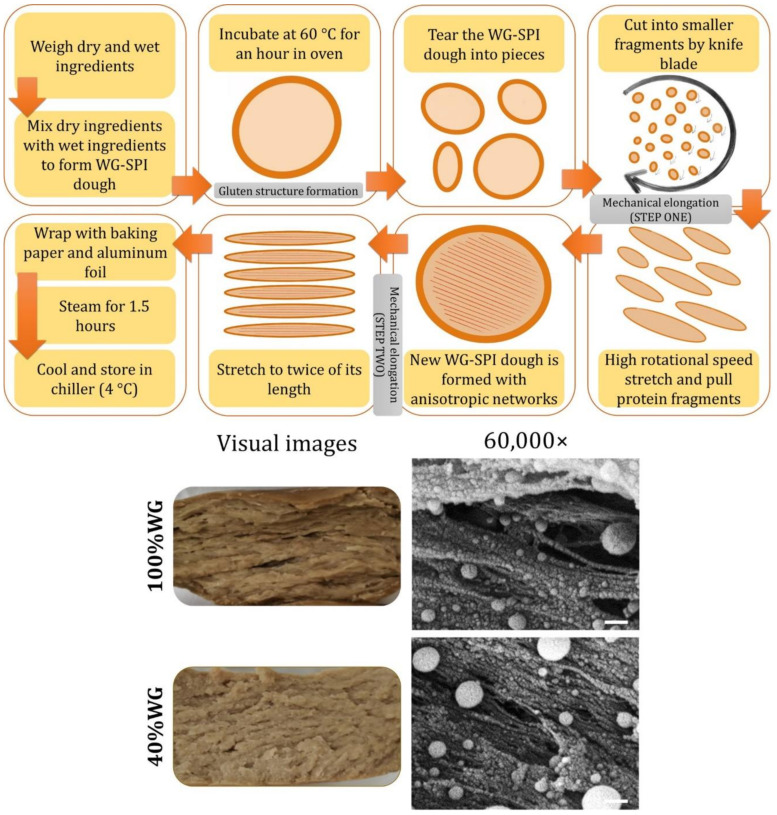
Schematic illustration of the mechanical elongation method in two steps to produce meat analogues and resultant microstructures. Adapted from [220] with permission.

The fibrous structuring of plant proteins using thermomechanical processing can be classified under two main principles. The first principle is based on phase separation within a multi-phase protein mixture [218,219]. The dispersed phase acquires a spherical droplet morphology under interfacial tension and undergoes deformation–elongation–solidification to form anisotropic structures in the direction of the applied shear [220,221]. A continuous protein phase possessing intrinsic properties (e.g., molecular composition, structure, and conformation) can acquire anisotropy during structuring, but does not have a dispersed phase that may impart structural anisotropy to the protein system. For instance, Krintiras et al. reported that soy protein isolate could be dispersed in a continuous wheat gluten matrix to form anisotropic structures, after shearing in a Couette cell [222]. In recent years, Mattice and Marangoni and Chiang et al. developed a more affordable technique using less sophisticated equipment, known as protein mechanical elongation methods [220,223]. These methods demonstrated potential in forming anisotropic structures using zein or wheat gluten by stretching and orientating the fine fibrils of the proteins (Figure 4). Further studies are recommended to understand the suitability (e.g., self-assembled networks) of protein ingredients, and to optimize the process conditions.

The second principle is based on the complex conformational changes and molecular interactions of the protein upon thermomechanical processing under high-moisture (40–80%) conditions such as extrusion [224]. As mentioned in the previous section, extrusion causes protein unfolding, with partial uncoiling of the secondary structure and a complete loss of tertiary structure [225]. Meanwhile, the hydrophobic and free SH groups that were initially buried inside the native protein are exposed. The shearing from the extrusion aligns the uncoiled protein molecules in the direction of flow, resulting in a three-dimensional network structure [218]. The texturization phase inside the cooling die initiates the solidification and formation of fibrous structure through the inter- and intra-molecular aggregation of the proteins. Many studies had been conducted on different sources of plant proteins. In recent years, Chiang et al. studied the effect of animal proteins (i.e., Maillard-reacted beef bone hydrolysate, MRP) with soy protein and wheat gluten to form extruded meat alternatives [226]. The inclusion of MRP not only increased the protein content of the meat alternatives, but also increased the sensory profiles of the meat alternatives. Further work could focus on using meaty flavors generated from plant proteins to increase the protein content and flavor profile of meat analogues.

#### 3.2.2. Formation of Gels

In addition to anisotropic fibrous structures, many foods are structured in the form of homogenous gels. These include yogurts, cheeses, tofu, tempeh, etc. Protein gelation occurs via various mechanisms. Globular protein gelation involves protein denaturation, aggregation, and network formation, whereas casein gels proceed via the aggregation of casein micelles. There are different methods to induce gel formation, including heat gelation, cold gelation by pH change (acidification, pH shift, fermentation), the addition of salts, enzymatic cross-linking, or pressure-induced gelation. Various types of gels can also be formed, such as hydrogels, oleogels (oil gels), aerogels and emulsion gels. Readers are referred to the excellent review by Cao and Mezzenga for a comprehensive examination of food gels [227].

**Figure 5:**
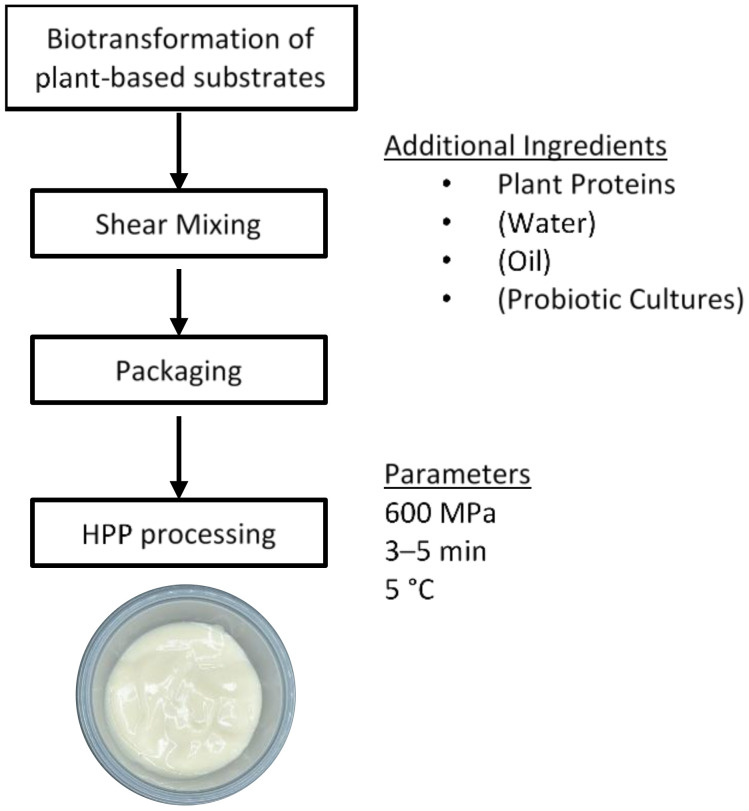
A proposed plant-based yogurt-making process using biotransformation for optimized flavor production and high-pressure processing (HPP) for consistent texture generation. From [236].

Plant protein gelation has long been utilized in traditional foods such as tofu and tempeh. One active area is the use of other plant proteins beside soy to produce traditional foods. Some examples include tofu and bean curds made from pea and various legumes [228,229,230]. Another active frontier is the development of plant-based yogurt and cheese analogues [231]. The wide variety of cheese styles with different textures and melt-stretch properties require different approaches to create. Some workers incorporate both tofu- and cheese-making steps involving the coagulation, pressing and fermentation of curds [232]. A recent study explored the use of zein to provide stretchability in plant-based cheese [233]. Traditional methods may not be optimal for plant protein ingredients; therefore, this presents opportunities to rethink the processes. For example, plant-based yogurt products presently adopt the traditional fermentation of plant-based milks [234]. Often, the acidified plant protein gels are weak and experience phase separation [235]. Sim et al. demonstrated a novel approach to structure plant-based yogurts using high-pressure processing [236], enabling separate operations to generate flavor and texture (Figure 5). In addition to creating plant-based animal alternatives, new product categories and unit operations should be explored.

Notably, most traditional foods structured by proteins are in the form of either hydrogels or emulsion gels. This is unsurprising, because proteins tend to have biological activity only in aqueous environments. In the design of future plant-based foods, a main challenge is mimicking the texture and mouthfeel of animal-based fats, because most plant-based lipids exist in the form of liquid oils. Solid fats also contain saturated fats of which excessive consumption has been associated with elevated cardiovascular disease risk and could lead to other health complications [237]. In contrast, oleogels have been found to reduce postprandial insulinemia and lipidemia [238,239,240,241]. By structuring plant oils into oleogels, plant-based lipids could be made to behave similarly to animal fat, for example, in plant-based meats [242]. The behavior of oleogels in structured plant proteins will be an important area of study, for example, to develop unique marbling in high-value meat analogues such as Wagyu beef. Proteins do not easily form networks in oil; therefore, it is challenging to use proteins as oil structurants. As such, most approaches use indirect methods such as foam-templated, emulsion-templated, hydrogel-templated, and solvent exchange procedures [243]. More work is needed on protein-based oleogels, especially using plant proteins.

### 3.3. Flavors Generated from Plant Proteins

Flavor is one of the sensory attributes that affects a consumer’s eating quality and food purchasing decision. Numerous studies on meaty flavor chemistry have discovered thousands of volatile compounds from meat or model systems consisting of meat ingredients. Due to the rising trend in alternative proteins, there is interest in developing this meaty flavor from non-meat sources such as plant proteins. These meaty flavors can be generated via the Maillard reaction, a process whereby free amino compounds (e.g., amino acids or peptides) and reducing sugars (e.g., pentoses or hexoses) are reacted together under specific conditions to produce melanoidins [244]. The plant proteins are broken down into amino acids and peptides through enzymatic hydrolysis to generate these meaty flavors.

The most abundant flavor compounds formed during the Maillard reaction are aliphatic aldehydes, ketones, diketones, and lower fatty acids [245]. However, heterocyclic compounds containing oxygen, nitrogen, sulfur, or combinations of these atoms are much more numerous and play a significant role in the flavor development of thermally processed foods. The development of a meaty flavor is often influenced by reacting sulfur-containing amino acids (e.g., cysteine) with reducing sugars, where pentoses such as ribose or xylose are preferably used [246]. The chemical reaction between cysteine and reducing sugars is believed to be the main pathway for the formation of meaty flavor for most food products. The dicarbonyl compounds formed during the Maillard reaction catalyze the Strecker degradation of cysteine to generate mercaptoacetaldehyde, acetaldehyde and hydrogen sulfide as the primary degradation products [247]. These Strecker degradation products then start a series of reactions that lead to the formation of meaty flavor compounds.

There have been published reports using several plant proteins to generate meaty flavors such as pea protein [248], quinoa protein [249], flaxseed protein [250], soybean protein [251,252], etc. Xylose was widely reported as the reducing sugar used for the Maillard reaction, except for a combination of sugars (ribose, xylose, arabinose, fructose, glucose and galactose) used by Zhou et al. when reacting with pea protein hydrolysates [248]. Based on the gas chromatography–mass spectroscopy analysis, several aroma compounds such as furans, pyrazines, ketones, aldehydes, and others were detected from the Maillard reaction products (MRPs). Both Wei et al. [250] and Fadel et al. [251] reported the identification of 2-methyl-3-furanthiol, an odorant compound characterized with a meaty, sweet and sulfurous aroma in the MRPs [244]. This compound was formed by the Maillard reaction of cysteine and reducing sugar in a model system. However, these authors also reported the addition of sulfur-containing compounds such as cysteine, taurine and thiamine together with the protein hydrolysates and reducing sugars in heat treatment for the Maillard reaction [248,249,250,251,252]. Further work could be conducted to avoid these sulfur-containing compounds and only use the free amino acids or peptides from the plant protein hydrolysates to react with the reducing sugars.

### 3.4. Nutrition as a Compass

Since the 1970s, nutritional scientists have claimed the existence of a “protein gap”. With the projected increase in population, providing sufficient protein for human health remains a topic of considerable importance. With an increasing demand for plant-based proteins, it is important to develop science and technology that will conserve both the functionality and nutrition of plant proteins. Historically, protein quality has been measured using either animals or humans. For example, net protein utilization (NPU) is a measure of the protein retained as a fraction of the protein consumed. Protein quality is dependent on digestibility (D) and biological value (BV); thus, NPU is a single value that represents both D and BV. Due to the procedure being onerous and time-consuming, it is customary to now use the protein digestibility-corrected amino acid score (PDCAAS) and digestible indispensable amino acid score (DIAAS). PDCAAS and DIAAS are used to measure the protein digestible quality based on both human amino acid requirements and digestible quality. PDCAAS is measured as the product of a dietary protein’s amino acid score to its actual fecal nitrogen digestibility (level of nitrogen excreted in feces relative to the level ingested). In contrast to the PDCAAS, DIAAS utilizes the ileal digestibility coefficients of each amino acid instead of the actual fecal nitrogen digestibility of the protein, to estimate the true ileal digestibility of the essential amino acids [253]. The essential amino acid content, protein digestibility, net protein consumption, biological value, and protein digestibility-corrected amino acid score all contribute to animal and plant protein’s nutritional quality (PDCASS, [254]). Animal proteins are often recommended to meet dietary protein needs at a modest caloric load and are considered of higher protein quality when compared to plant sources. Animal proteins tend to contain a good balance of all the essential and non-essential amino acids, whereas plant proteins are deficient or limited in one or more essential nutrients [255]. Plant proteins are said to be nutritionally insufficient due to a lack of certain necessary amino acids and are gastro-intestinally less bioavailable or less digestible when compared to animal proteins. Most grain proteins, for example, are low in lysine, whereas pulse proteins are low in methionine, cysteine, and tryptophan. The highest PDCAAS/DIAAS scores are for animal proteins (>0.9), whereas the plant proteins often have lower scores ranging from 0.4 to 0.9, as shown in Table 1 [255,256]. There are three main factors responsible for the reduced digestibility of plant proteins: (1) the presence of anti-nutrients such as phytates and trypsin inhibitors that interfere with digestion and absorption of proteins [257]; (2) the structural differences between animal and plant proteins, i.e., plant proteins contain fewer α-helixes than animal proteins and more β-sheet structures, which facilitates protein aggregates [258]; and (3) the presence of dietary fibers that lower proteolytic digestibility in plants [259].

Apart from the bioavailability of proteins during gastrointestinal digestion, the nutritional value of protein sources is influenced by the rate/kinetics of protein digestion. Proteins are classified as slow proteins and fast proteins based on the kinetics of protein digestion [260,261]. Whey proteins are digested more quickly and absorbed in the body than native micellar casein because caseins coagulate with digestives enzymes resulting in slower digestion and subsequent absorption [260]. Soy proteins are absorbed more quickly than casein and more slowly than whey proteins [262]. The muscle protein fractional synthetic rates of whey, soy and casein are 0.091, 0.078 and 0.047 %/h, respectively [262]. However, in another study, the rate of postprandial muscle protein synthesis of soy proteins did not rise as much as it did after consuming whey proteins [263] which could be related to amino acid composition, i.e., low content of leucine which increases protein synthesis while inhibiting protein breakdown [264].

There are few strategies to improve the quality of plant-based proteins for human consumption. Firstly, to increase the protein’s nutritional quality to PDCAAS 1.00, which is comparable to animal-derived foods, the anti-nutrients are removed from the isolated plant-based proteins (e.g., soy). Secondly, increasing the amount of plant-based protein consumed per meal is predicted to effectively compensate for their reduced anabolic response in comparison to animal protein [265]. Having said that, plant proteins would not result in a similar anabolic response when compared to animal sources with these strategies, because removing the anti-nutrients or increasing the intake of plant-based proteins may not increase the essential amino acid content: mainly leucine [17,257]. Therefore, the consumption of wheat protein with the equivalent proportion of leucine content to that of whey protein has been shown to increase rates of postprandial muscle protein synthesis [266]. Thirdly, fortifying plant proteins with amino acids required to perform protein synthesis is another strategy to improve the nutritional quality of the plant protein. A study has shown that fortifying soy proteins with leucine, isoleucine, and valine increased whole-body protein synthesis [267]. Additionally, blending the right combination of plant-based proteins will provide a complex source of amino acids [268] which can increase the nutritional quality of plant proteins. According to Day (2013), the individual PDCASS value of peas and rice are low; however, the combination of peas and rice together can increase the PDCASS value to 1.00 [269]. Incorporation/fortification or modification of plant proteins with an effective strategy can enhance the nutritional quality of plant-based proteins, opening new growth opportunities for the food industry.

## 4. Conclusions

This review has presented a roadmap to accelerate plant protein science and technology, focusing on plant protein ingredient development and the creation of delicious and nutritious plant-based future foods. The areas for further improvement include plant protein extraction, fractionation, and modification. More research is also needed in understanding plant protein–polysaccharide interactions, developing different structuring techniques, incorporating plant protein-generated flavors, and improving plant protein nutritional value. An area that needs future attention is the potential impact that different forms of fractionation and improved functionality may have on its nutritional quality. Finally, although the focus has been on plant proteins, it is vital to note that we usually eat whole foods and not individual ingredients; hence, other components that make up future foods will also be needed to be considered.

## Figures and Tables

**Table 1:** PDCAAS and DIAAS for protein fractions and foods. Adapted from [256].

Food	PDCAAS	DIAAS	Limiting Amino Acids
Soy protein isolate	0.98	0.90	Met + Cys
Pea protein isolate	0.89	0.82	Met + Cys
Rice protein concentrate	0.42	0.37	Lys
Cooked Peas	0.60	0.58	Met + Cys
Cooked Rice	0.62	0.59	Lys
Almonds	0.39	0.40	Lys
Chickpeas	0.74	0.83	Met + Cys
Tofu	0.56	0.52	Met + Cys
Whole Milk	1.00	1.14	Met + Cys

PDCAAS—protein digestibility-corrected amino acid score; DIAAS—digestible indispensable amino acid score; Met—methionine; Cys—cysteine; Lys—Lysine.
